# Performance of Narrow Band Wide Area Networks with Gateway Diversity

**DOI:** 10.3390/s22228831

**Published:** 2022-11-15

**Authors:** Başak Can, Bora Karaoğlu, Uttam Bhat, Muhammed Faruk Gencel, Thomas Chen

**Affiliations:** 1 Amazon Lab126, 1100 Enterprise Way, Sunnyvale, CA 94089, USA; 2 Amazon Lab126, Ciyun Rd., East Dist., Hsinchu City 300196, Taiwan

**Keywords:** coverage area, gateway, gateway diversity, IoT, LoRa, LPWAN, PER, PSR, RSSI, SNR

## Abstract

This paper quantifies the coverage area of Low-Power Wide-Area Networks (LPWAN) for Packet Success Rates (PSR) above 85%, where acceptable Quality of Service (QoS) can be achieved. The network consists of battery-operated end-nodes (ENs) and multiple stationary gateways (GWs). We consider asynchronous communication that uses ALOHA-based random channel access. Each transmission from the ENs can be received by multiple GWs. Such spatial diversity results in favorable Signal-to-Noise ratios (SNR). The LoRa modulation is assumed and its specific features, such as IQ inversion, further contribute to decreasing the impact of interference. An increase in the GW density improves network performance, which allows support for a larger density of end-nodes as well as increasing the coverage area. Our simulation results show that a suburban area of up to 1.44 km^2^ can be covered with five GWs with up to fifty end-nodes with a PSR greater than 86%.

## 1. Introduction

Low-Power Wide-Area Networks (LPWAN) enable the Internet of Things (IoT) applications and hardware, such as smart meters, supply chain and logistics trackers, smart home/city sensors for automation, agricultural sensors, retail store sensors, asset/pet trackers, parking sensors, environmental sensors, healthcare sensors, safety and security sensors, remote controls, etc. The majority of the aforementioned IoT applications require battery operation with very low application data rate requirements, making long-range and low-power consuming modulation and coding schemes a viable choice. The use of low bandwidths and sub-GHz carrier frequencies, along with using constant envelope modulations in turn, reduces the hardware cost of the IoT sensors significantly, allowing wide adoption [1,2,3]. The sensors should be designed with battery operation in mind and hence very low bandwidths, such as 125 kHz, 250 kHz, and 500 kHz that are typically used with constant envelope modulation schemes. For example, the LoRa modulation has been a very popular choice as it can work under noise, thanks to the processing gain provided by the chirped spread spectrum (CSS) technique. The aforementioned processing gain per bit allows the LoRa modulation at the PHY layer to achieve very good sensitivity levels. This increases the range of the wireless link at the cost of increased airtime with increasing processing gain, resulting in lower bitrates along with susceptibility to channel variations due to mobility. 

Gateways (GWs) are hardware entities that have access to the internet/cloud that are the building blocks of the LPWAN. They typically have access to wall power or solar power and do not operate on a battery. Furthermore, they can be installed outside of homes or garages, allowing them to reach a better coverage area. Hence, GWs are typically more sophisticated and more powerful than end-nodes, allowing them to process data from multiple sensors. End-nodes refer to the HW sensors that operate on a battery. They rely on a GW to access the cloud to send sensory data. The Uplink (UL) refers to the traffic initiated by an end-node that is received by a GW. Similarly, Downlink (DL) refers to the traffic sent by a GW to end-nodes. Typically, the UL is the bottleneck in such WAN as multiple end-nodes have to content for successful transmission to the nearest GW and suffer from collisions. Furthermore, it is easier to establish coordination among GWs (for DL) with high-bandwidth cloud links. 

**Figure 1:**
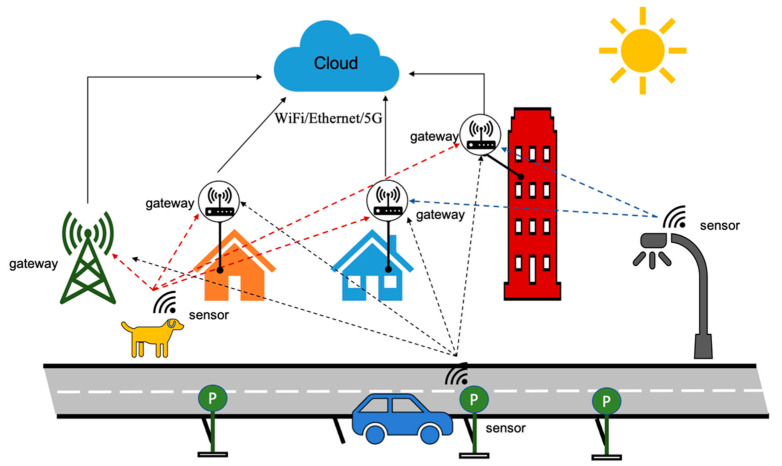
LPWAN with GW diversity.

The operation of the LPWAN network is depicted in Figure 1 as an example. The sensor (as an end-node (EN)) is trying to access a GW, e.g., smart metering purposes, which requires very low bitrates such as 2 kbps. It can access the cloud via the closest neighbor’s GW or via the user’s own GW which may not necessarily have the closest proximity to the sensor. This is referred to as GW diversity, where multiple GWs can pick up an ENs packet and pass it on to the cloud server for being sent to the application layer. In the later sections of this paper, the simulation results show the benefits of such GW diversity.

In [4], the authors study the performance of LPWAN with message replication and multiple receive antennas. The authors provide an optimum number of message copies for different data rates and show improvements with time and antenna diversity in the UL. In [5], the authors perform experimental results for UL with GW diversity in an urban area. The coverage is defined as distances to the GW where at least one packet was received successfully out of many packets. However, this causes the Packet Success Rate (PSR) target to be set to only 1% at the declared range if only one packet was received out of one hundred, which does not provide a stable link. Hence, such an assumption causes overpromising results for range as it does not consider maintaining a reliable PSR per link. In this paper, the coverage area is defined for a given PSR target. Unlike existing literature which mainly analyses the UL performance [6], this paper presents not only the UL performance, but also the DL performance which is an essential part of the network. In UL, multiple GWs can listen to the packets from the ENs, hence providing spatial diversity. In DL, only one of the GWs responds to an EN. This paper presents the performance evaluation to determine the number of GWs to achieve a certain PSR target in a given coverage area. This paper also evaluates the coverage area that can be achieved under interference from multiple end-nodes and multiple GWs while considering packet collisions. 

The paper is organized as follows. In Section 2, the principles of the LoRa modulation are introduced, followed by the illustration of the benefits of IQ inversion for the LoRa modulation. The system model for channel access, interference, and path loss can be found in Section 3. Finally, the numerical results and conclusions of this work are presented in Section 4 and Section 5, respectively.

## 2. Principles of the Lora Modulation

The principles of the LoRa modulation have been analyzed in detail in the literature along with detailed analytical models [7,8,9]. Only the key parameters/performance indicators (KPIs) of this modulation are presented in this section. Let BW represent the bandwidth used per channel. Let SF represent the Spreading Factor that is being used per channel and let CR represent the Forward Error Correction (FEC) code rate. With these definitions, the LoRa symbol duration, chip duration, bitrate, and chirp rate can be calculated as follows:

(1) $$\mathrm{Chip}\;\mathrm{duration:}\;T_{c}\;=\frac{1}{BW}\;[s]$$

(2) $$\mathrm{Symbol}\;\mathrm{duration:}\;\;T_{s}=2^{SF}\;T_{c}\;\;[s]$$

(3) $$\mathrm{Bitrate:}\;\rho =CR\times SF\times \frac{1}{T_{s}}\;[b/s]$$

(4) $$\mathrm{Chirp}\;\mathrm{rate:}\;\mu =BW\times \frac{1}{T_{s}}=\frac{BW^{2}}{2^{SF}}\;[\mathrm{Hz}/s]$$

(5) $$\mathrm{The}\;\mathrm{Bit}\;\mathrm{Energy}\text{-}\mathrm{to}\text{-}\mathrm{Noise}\;\mathrm{ratio:}\;\frac{E_{b}}{N_{0}}=\frac{2^{SF}}{SF}\times SNR$$

The SF determines the number of bits per symbol. Equation (5) shows the required SNR in relation to the bit energy-to-noise ratio with the processing gain provided by the CSS signal processing. Thanks to this processing gain denoted by $\frac{2^{SF}}{SF}$, the system is able to recover signals below the thermal noise floor. Hence, sensitivity can be achieved with an SNR < 0 dB [7]. For example, the processing gain with SF-11 and SF-8 is 22.6 dB and 15 dB, respectively. 

The un-modulated transmit signal based on up-chirps with a chirp rate of μ  can simply be defined as:

(6) $$s\left(t\right)=V\times e^{j2\pi \left(\frac{\mu t}{2}-\frac{BW}{2}\right)t}\times e^{\left(j2\pi f_{c}t+\theta \right)},\;0\leq t\leq T_{s}$$

**Figure 2:**
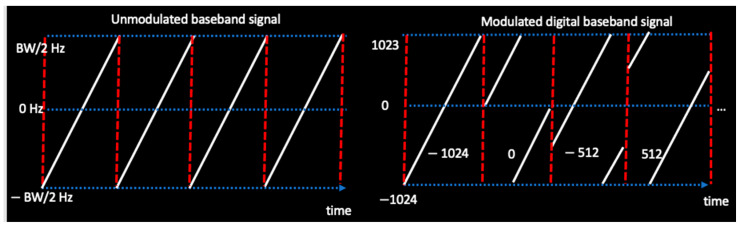
The LoRa modulation example in frequency versus time domain with SF-11.

The term *V* represents the desired signal magnitude, which is a constant since the modulation is carried in the frequency domain. Such modulations are referred to as constant envelop modulations. This makes the LoRa appealing especially for being able to work with non-linear and efficient power amplifiers. The term $f_{c}$ represents the carrier frequency. The signal is then modulated by the cyclic shift on the base chirp signal containing $2^{SF}$ chips spanning from $-\frac{BW}{2}$ Hz to $\frac{BW}{2}$ Hz. The base chirp, with slope μ, wraps around in time within these bounds. When modulated, there are essentially $2^{SF}$ possible starting points for the chirp to start and eventually wrap around itself. Where the chirp starts (from $-2^{SF-1}$ and up to $2^{SF-1}-1$) carriers the information bits (SF bits per symbol). This is illustrated in Figure 2 for SF-11, with 2048 chips per symbol. The illustrated mapping can be improved with gray coding. 

**Figure 3:**
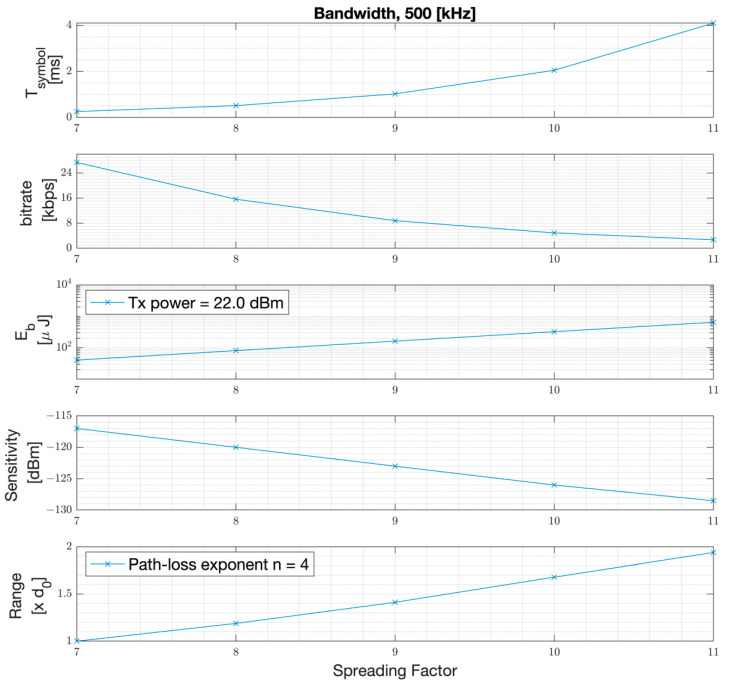
PHY symbol duration, PHY bitrate, energy per bit, sensitivity, and range versus SF.

Based on Equations (1)–(4), the PHY symbol duration, PHY bitrate, energy per bit, the measured sensitivity, and range versus the spreading factor SF are plotted in Figure 3. As the results show, the conducted sensitivity can be improved by 11.5 dB by changing the spreading factor from SF = 7 to SF = 11. Assuming an outdoor path loss exponent of n = 4, this achieves a range improvement of approximately 100% in meters. However, such improvement comes at an exponential increase in symbol duration and hence, a significant drop in bitrate. This makes the LoRa modulation suitable for applications requiring low bitrates, (e.g., 2 kbps PHY rate with SF-11 with a code rate of 4/5) and low mobility as the channel should be static during the course of much longer packet durations. 

**Figure 4:**
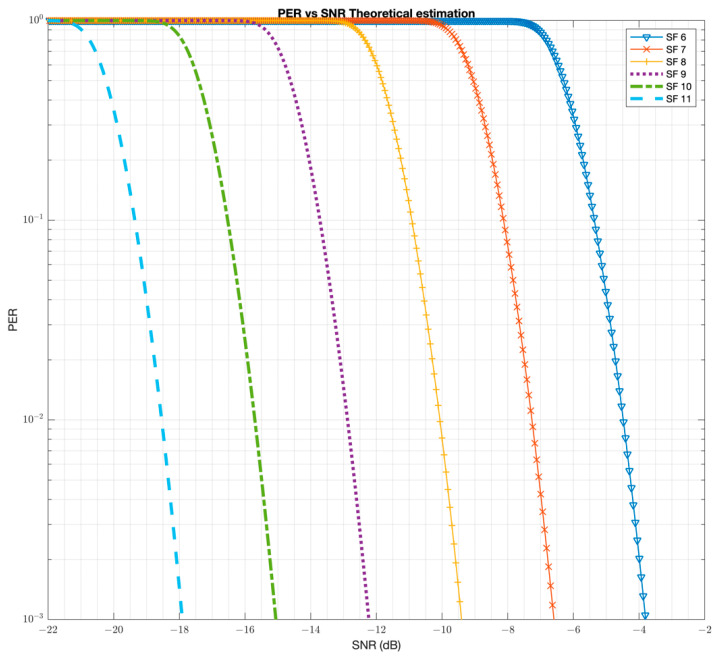
The PER estimate with the LoRa modulation with 64 bytes payload.

In order to understand the capabilities and limitations of the LoRa modulation, extensive simulations have been obtained and compared to measurements for benchmarking the simulator. The simulator for the LoRa modulation is originally obtained from [10]. That simulator did not have the capability of injecting Additive White Gaussian Noise (AWGN). The AWGN analysis capability has been added to the simulator. This helped in comparing the simulation results to the data sheet for sensitivity. The laboratory measurements are obtained with an SX1261/2 chipset in a conducted setup and are compared to those in the datasheet as well [11]. Additionally, the Bit Error Rate (BER) can be estimated as $BER=0.5\times Q\left(\sqrt{SNR\;2^{SF+1}}-\sqrt{1.386\times SF+1.154}\right)\;$ [12]. Then, the Symbol Error Rate (SER) and PER can be calculated as $SER=\frac{2^{SF}-1}{2^{SF-1}}\times BER$ and $PER=1-{\left(1-SER\right)}^{N_{symbols}}$. The PER estimation based on these equations is presented in Figure 4.

The simulation results (Figure 4) versus datasheet numbers are presented in Table 1. The results show that the simulation results are within 1 dB accuracy to that published in the datasheet (Table 6.1 in [11]), and hence it is a good tool to use for further performance evaluation. 

**Figure 5:**
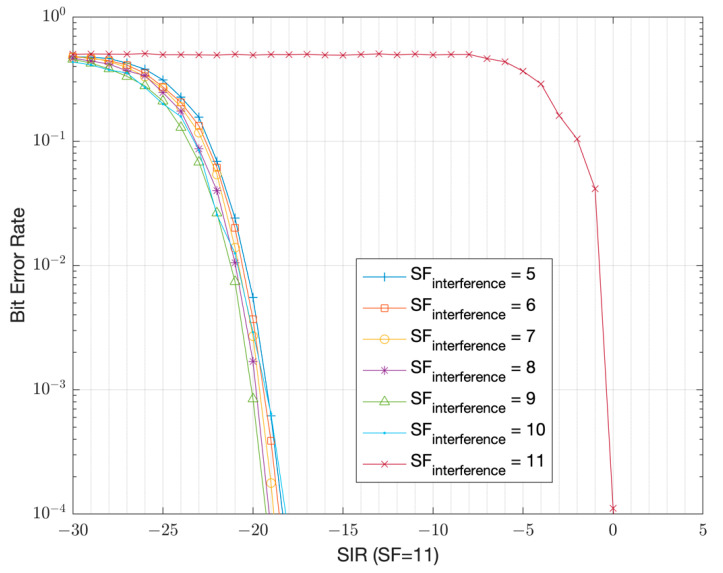
BER versus SIR without IQ inversion with SF-11 (500 kHz BW) [9].

**Figure 6:**
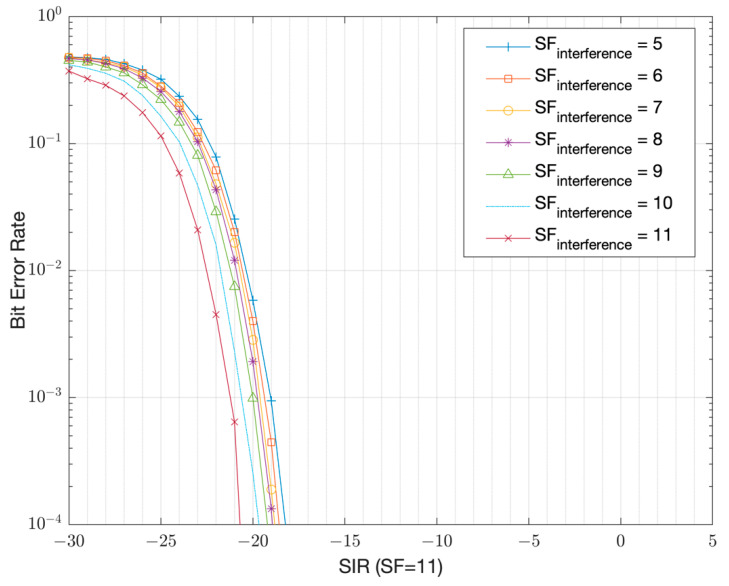
BER versus SIR with IQ inversion with SF-11 (500 kHz bandwidth).

The simulation results that show the resilience to interference is presented in Figure 5 in the form of the Signal-to-Interference Ratio (SIR), assuming 500 kHz bandwidth. As the results versus the spreading factor show, the signals with the same chirpiness (the same chirp rate as shown by Equation (4)) are non-orthogonal to each other [8,9]. For the same chirp rate, the orthogonality can be achieved by using IQ inversion, (i.e., complex conjugation) of the main chirp used for modulation and demodulation. The IQ inversion means that instead of up-chirping the symbols at the modulator, down-chirping is used and the receivers start demodulating with the opposite up-chirps. The results in Figure 5 and Figure 6 show that close to orthogonal performance can be achieved in between two LoRa signals despite using the same chirp rate as long, as they use the opposite chirp direction. For example, if GWs use the opposite chirp direction for DL versus the end-nodes in the UL, the effects of interference between UL and DL transmissions can be reduced significantly. For example, a SIR ≥ −20.7 dB is sufficient instead of a SIR ≥ 0 dB with SF-11 for a BER target of $10^{-4}$ (Figure 5 and Figure 6). Simulation results further show that a worst-case SIR of at least 0 dB is required within UL transmissions to achieve a Bit Error Rate (BER) target of $10^{-4}$ when using SF = 11 among the end-nodes (Figure 5). A collision does not always result in a packet loss as long as the SIR is above the thresholds presented in Table 2. This table is prepared from the BER versus SIR simulation curves and extracted at a 1% Packet Error Rate (PER) target, corresponding to a BER target of 10^−4^ (Table 1).

## 3. System Model

In this section, the channel access and interference model along with the path loss model are presented which are used in our NS-3 simulations.

(A) Channel Access

In this section, the channel access scheme that is used throughout the analysis and simulations are presented. The LPWAN module follows an ALOHA-based sporadic channel access model in the UL with spatial diversity among the GWs, where all the GWs listen to the UL traffic at all times [13,14]. The network architecture follows a star topology. When a given GW can successfully decode an ENs UL packet, it passes it up to the cloud server to be sent to the application layer. The cloud server processes multiple copies of the same packet on a first come, first served basis and discards duplicates sent from multiple GWs. Coherent combining of the packets from the GWs as presented in [15] is beyond the scope of this paper. 

The underlying PHY modulation is LoRa but the upper layer channel access is different than the LoRaWAN protocol. The ENs use a single SF and a single channel. The GW diversity concept is similar to LoRaWAN, but our system model assumes single radio gateways unlike LoRaWAN, which can support multiple channels and data rates simultaneously. The best GW is determined by the cloud server based on the strongest UL RSSI among multiple GWs receiving a UL packet from a given EN, which best GW serves for the DL direction for that EN. 

By allowing the end-nodes to transmit when they have traffic, their transceivers can go to sleep to save energy, rather than periodically waking up to search for a transmit opportunity or a beacon from the GW. In the DL, the GW aligns its transmission to the periodic receive windows of the ENs after timing a handshake between the GW and the EN. The expected network load is sporadic and light enough to make the energy consumption on end-nodes the main consideration rather than the bandwidth efficiency. Moreover, the lack of overhead and decentralized channel access decisions are well-suited for applications with battery operation. The drawback of this scheme, however, is the interference in the UL towards the GW, as there is no coordinated channel access mechanism.

(B) Interference Model

**Figure 7:**
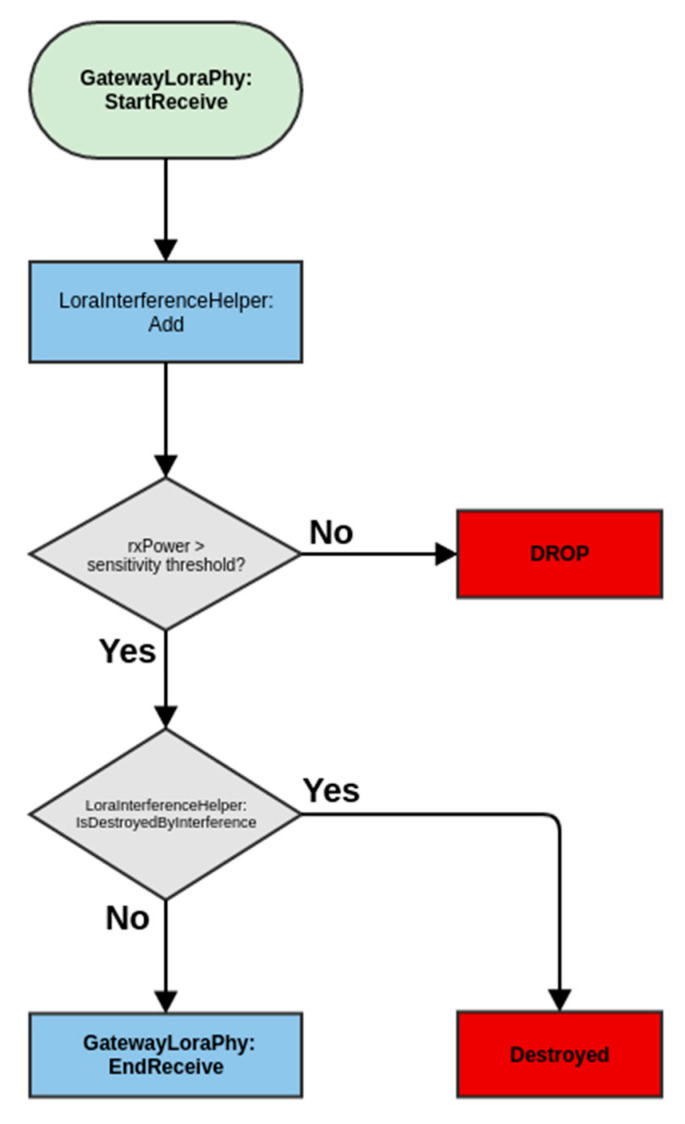
The interference model in the LPWAN module (illustration of procedures 4.2 and 4.3 in [16]).

In this section, the interference model is presented. Packets can potentially collide due to an un-coordinated ALOHA-based channel access scheme [14]. To simplify the EN operation, the UL and the DL share the same RF channel (902 MHz) and use the same spreading factor. The chirp symbols in the DL transmissions are IQ inverted, thereby providing the orthogonality with respect to UL transmissions. For such a scenario, as the results in Figure 5 and Figure 6 show, the worst-case SIR threshold that is required to achieve a BER target of 10^−4^ is SIR_th_ = 0 dB within end-nodes in the UL direction and SIR_th_= −20.7 dB in between the UL and DL traffic thanks to IQ inversion. These thresholds correspond when the LoRa modulation is used with an SF = 11 and with a 500 kHz bandwidth. During the analysis for the system PER, any collisions occurring with a SIR < SIR_th_ are assumed to cause a packet loss. This is illustrated in Figure 7. The WAN operates based on the LoRa modulation with either SF-11 at ~2 kbps PHY bitrate assuming a code rate of 4/5, or with SF-8 at ~16 kbps PHY bitrate.

(C) Path loss Model

**Figure 8:**
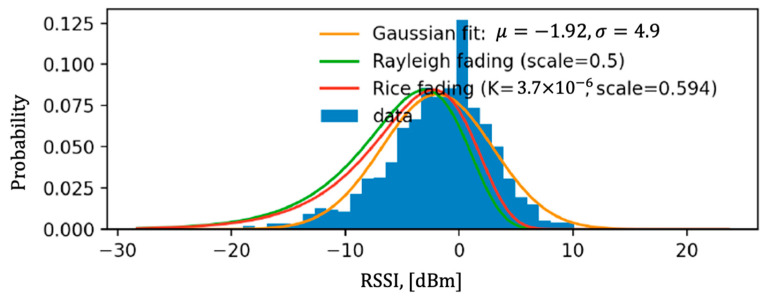
RSSI samples in dBm and their histogram (samples shifted to center around 0).

**Figure 9:**
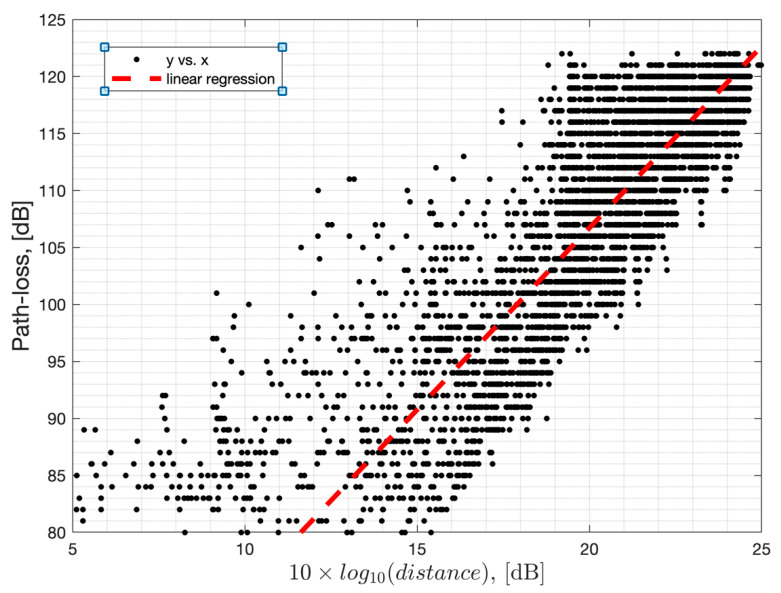
Path loss measurement versus log distance from the GW in a suburban neighborhood in Mountain View, CA, USA.

In this section, the path loss model is presented for the US 902–928 MHz ISM band. We define the path loss (in dB) including the shadow fading as follows:

PL(d,f) = PL_0_(d_0_, f_0_) + 10log_10_(d/d_0_)^n^ + 20log_10_(f/f_0_) + X(0,σ), [dB] (7)

where PL_0_(d_0_, f_0_) is the path loss at reference distance d_o_, and reference channel frequency, f_0_. The term n represents the path loss exponent, d is the distance between the GW and the EN, and f is the channel frequency. The term X(0,σ) (in dB) is a random variable that represents the large-scale shadow fading effects. Our field measurements show that it has a log-normal distribution and shows a standard deviation of σ = 5 dB ( Figure 8). Please note that these parameters depend on the environment that they are measured and hence can be different in different terrains or cities. Path loss is calculated based on the difference between the measured Received Signal Strength Indicator (RSSI) reported by the EN and the Transmit Power (TxP) at the GW, where TxP = 22 dBm. Both the EN and the GW were at 1.4 m from the ground. The path loss measurements versus the log-distance are presented in Figure 9, which are measured with an Evaluation Kit (EVK) with an SX-1262 chipset. The variation in the path loss around a given distance is due to shadow fading which changes versus time. With linear regression applied to the random path loss measurements, we estimate the mean path loss exponent to be n = 3.2, with 95% confidence bounds of [3.14, 3.2]. Additional HW losses of 14.5 dB and an environmental in-band interference level of 6 dB were added to the overall path loss based on field measurements. HW losses refer to the overall end-to-end losses caused by Tx and Rx antenna efficiencies, RF front-end losses, and chipset-to-chipset variations.

## 4. Numerical Results

This section presents numerical results derived from a simulation study on a Network Simulator-3 (NS-3) with the ns-3.33 version [16,17,18]. The simulator is modified to match the system model presented in Section 3. The PSR and PER are related as follows: PSR = 1-PER. The end-nodes and gateways are randomly positioned within the simulated area per iteration and the average PSR, along with its distribution, is calculated across all the iterations. Each node generates on average one packet per two minutes. 

**Figure 10:**
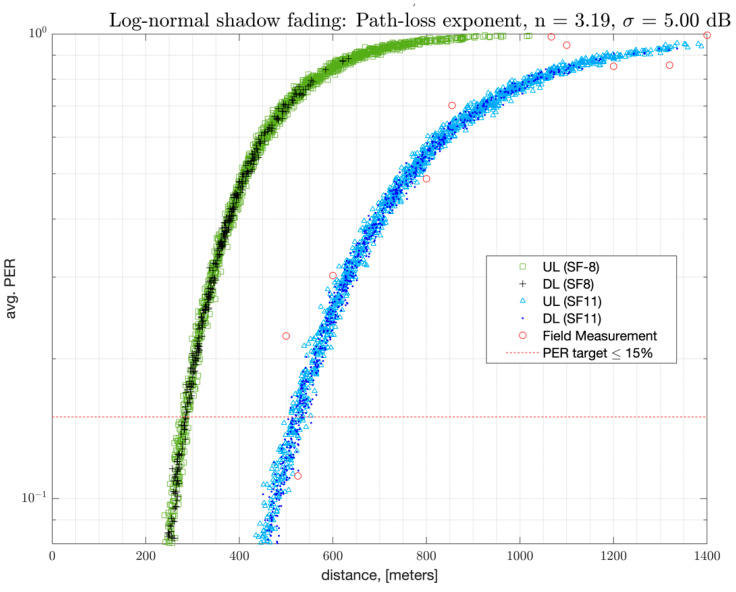
PER versus distance to the GW in suburban terrain (1 GW, 1 EN, and 44 bytes payload).

In order to illustrate the link budget with the LoRa modulation, the average PER versus distance between one GW and one end-node is presented in Figure 10. A link-level range of 524 m can be achieved with an average PSR, (i.e., 1-PER) of at least 85% for 99 percent of the iterations with SF-11. Such PSR is referred to as the Top Percentile 99 (TP-99) PSR, which is derived from the empirical cumulative distribution function (eCDF) of the PSR across iterations. Such TP-99 PER versus distance obtained from the field measurements are also added to the plot to illustrate that the developed model predicts the coverage area well. The TP-99 results from the field are obtained over the worst-case interference levels across several sites at the same distance to the GW. Results are measured with static locations in the Westchester, CA suburban area with 1000 packets per site, where multiple sites are tested per the distance to the GW. Such an impact from interference derived from the field measurements is added to the link budget in the NS-3 simulator as well. The field measurements are obtained with an EVK to EVK link with an SX-1262 chipset.

**Figure 11:**
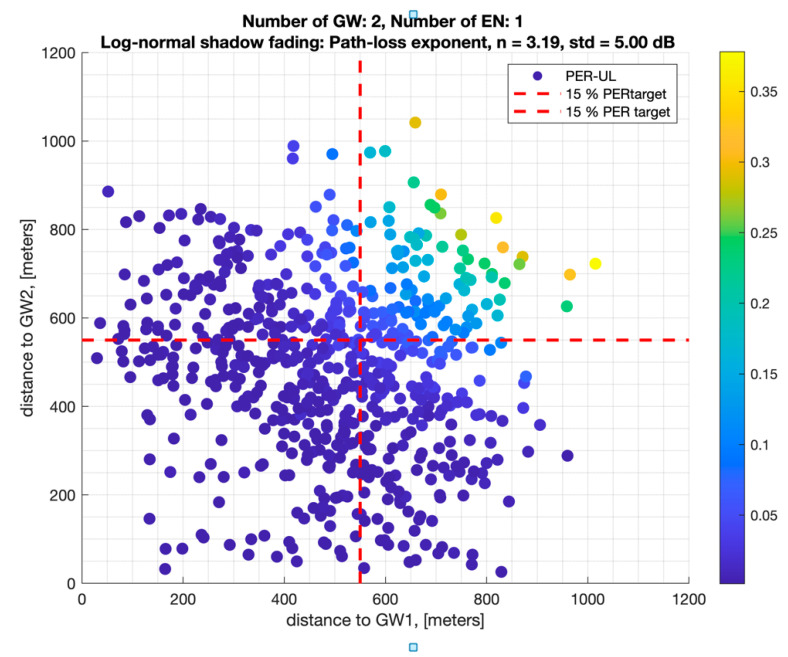
PER versus distance to each GW for a single EN in suburban terrain/with GW diversity.

When there are two gateways serving a single node, the EN packets can be forwarded by either of the two GWs. Figure 11 illustrates the average UL PER of an EN with respect to its distance to each of the GWs. We observe that an EN with a 524 m distance to either GW has a PSR > 97%, which is significantly better than a single GW. Similarly, the ENs that are more than 524 m away from one of the GWs can still achieve a PSR > 85%. This phenomenon is called GW diversity, which helps GWs together cover regions that are beyond their individual coverage area. 

**Figure 12:**
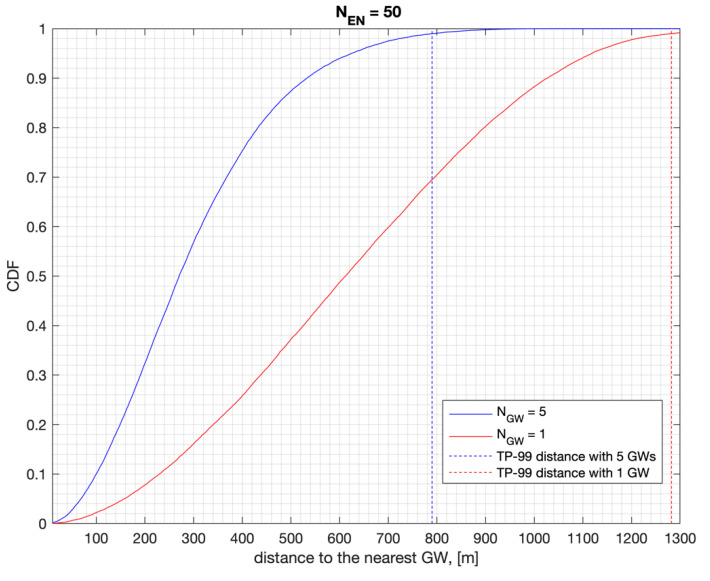
The CDF of distance to the nearest GW with 50 ENs.

Even though GW diversity is effective against path loss, it is susceptible to interference and is expected to lead to reduced returns in high-interference deployments. Its impact on the coverage area with an increase in the number of end-nodes in the network is illustrated using the results obtained from the NS-3 simulation, and are presented in Table 3. It is observed that as the end-nodes in the network increase, the interference in the network increase in turn, negatively impacting PER. The devices that are towards the edge of the network range become susceptible to interference from end-nodes that are closer to the gateway. Furthermore, as the simulation region is expanded, the nodes suffer from increased path loss. We see that with fifty ENs and one GW distributed over a 1.44 km^2^ region, the TP-99 PSR drops to as low as 39%. In a similar scenario, in order for the TP-99 PSR to reach 85%, the number of GWs needs to be increased to five. The improvement in PSR is achieved due to GW diversity and thanks to the improved link margin for the devices, which were earlier at the edge of the network and now get a chance to be closer to another GW (Figure 12). The improvement in the coverage area is observed to increase to 1.44 km^2^ with SF-11 with a TP-99 PSR of 86%. Similar results are observed with SF-8, where an area of 0.42 km^2^ can be covered with a TP-99 PSR of 87%. The DL TP-99 PSR is slightly worse than UL PSR in a large area when GW diversity is used. That is because the best GW is decided during the UL transmissions, and that same GW is used for DL transmissions, where the shadow fading might change.

The coverage area results highly depend on the traffic load on the network as determined by the number of ENs, GWs, and packet rates. The CDF of the distance to the nearest GW is presented in Figure 12 and calculated across all the iterations for an area size of 1200^2^ square meters. The TP-99 distance, d, refers to the case where 99% of the iterations have a distance less than d meters to the nearest GW. This is when the CDF curve shows a value of 0.99 at distance d. This metric can be used to refer to 99% of the cases across all the possible cases for sufficiently large iterations. For 50 ENs served by a single GW, the TP-99 distance to the GW is 1.3 km. The TP-99 distance to the nearest GW drops to 790 m if there are at least five GWs serving the same area size. This improves the link budget significantly and results in a higher PSR in both the UL and the DL, as presented in Table 3.

## 5. Conclusions

The study in [19] illustrates the benefit of GW diversity in terms of improved PER with only two gateways measured in an indoor anechoic chamber. This study with realistic system parameters such as outdoor NLOS conditions with shadow fading shows that a coverage area of 1.44 km^2^ can be achieved with a TP-99 PSR ≥ 86%. This can be achieved with SF-11 in a suburban terrain type as long as there are multiple GWs providing GW diversity. This observation is made when the packet injection rate is sparse with one packet sent every other minute from the ENs. Such coverage area will shrink when the packet injection rate to the network is increased, causing more collisions in the UL. Although the coverage area size drops to 0.42 km^2^ when SF-8 is used, the PHY bitrate is increased by 5.8-fold for the same bandwidth. The LoRa modulation is best suited for stationary end-nodes due to the long packet durations. Future works based on this study may include, but are not limited to, the following: 1. Implement a mobility model and packet repetitions to the large scale simulator and re-assess the coverage area for mobile applications such as asset/pet tracking. 2. Assess system capacity versus packet injection rate of the network. 3. Develop a path loss and interference model for rural and dense urban terrain types and re-assess the coverage area. 

## Figures and Tables

**Table 1:** BER and PER simulation results versus datasheet for AWGN channel (64 bytes payload).

Spreading Factor	SNR at 1% *PER*, [dB], (Simulation)	*BER* (Simulation)	SNR at 1% *PER*, [dB], (Datasheet)	Simulation to Datasheet Delta, [dB]
6	−4.5	$10^{-4}$	−5	0.5
7	−7		−7.5	0.5
8	−9.9		−10	0.1
9	−12.75		−12.5	−0.25
10	−15.5		−15	−0.5
11	−18.4		−17.5	−0.9

**Table 2:** C/I (SIR) results versus SF at BER = 10^−4^ with 500 kHz bandwidth with 64 bytes payload.

C/I [dBc]		Interference (LoRa)						
	SF/BW (kHz)	5/500	6/500	7/500	8/500	9/500	10/500	11/500
Desired signal (LoRa)	5/500	1.26	−0.83	−2.1	−3	−3.6	−3.8	−4.3
		with IQ inversion						
		−3.1	−3.5	−3.6	−3.7	−3.9	−4.1	−4.6
	6/500	−4	1	−3.33	−4.8	−5.65	−6.2	−6.5
		with IQ inversion						
		−5.3	−5.8	−6.1	−6.4	−6.5	−6.7	−7
	7/500	−7.8	−6.6	0.8	−6	−7.7	−8.6	−9
		with IQ inversion						
		−7.8	−8.1	−8.7	−8.7	−9.1	−9.2	−9.6
	8/500	−10.6	−10.6	−9.4	0.54	−9	−10.7	−11.6
		with IQ inversion						
		−10.5	−10.6	−10.9	−11.6	−11.4	−12.1	−12
	9/500	−13	−13.5	−13.5	−12.2	0.36	−11.9	−13.7
		with IQ inversion						
		−13.1	−13.3	−13.5	−13.8	−14.6	−14.2	−15.1
	10/500	−15.7	−16	−16.2	−16.4	−15	0.14	−15
		with IQ inversion						
		−15.6	−15.9	−16.1	−16.3	−16.8	−17.7	−17.2
	11/500	−18.4	−18.5	−18.8	−19	−19.3	−18.2	0
		with IQ inversion						
		−18.2	−18.6	−18.8	−18.9	−19.2	−19.7	−20.7

**Table 3:** PSR versus number of nodes in suburban terrain type.

SF	# of ENs	# of GWs	Avg. PSR	Avg. PSR	TP-99 PSR ≥	TP-99 PSR ≥	Simulated Area Size [km^2^]	95% Confidence Interval on Avg. PSR [%]
			UL [%]	DL [%]	UL [%]	DL [%]		
11	1	1	98	98	85	85	0.24	±0.2
11	50	1	60	79	39	62	1.44	±0.8
11	50	5	95	95	90	86	1.44	±0.2
8	1	1	98	97	85	85	0.1	±0.5
8	50	1	65	79	40	60	0.42	±0.9
8	50	5	98	96	94	87	0.42	±0.2

## Data Availability

Not applicable.
